# Uncomplicated Diverticular Disease: Innate and Adaptive Immunity in Human Gut Mucosa before and after Rifaximin

**DOI:** 10.1155/2014/696812

**Published:** 2014-07-16

**Authors:** Rossella Cianci, Simona Frosali, Danilo Pagliari, Paola Cesaro, Lucio Petruzziello, Fabio Casciano, Raffaele Landolfi, Guido Costamagna, Franco Pandolfi

**Affiliations:** ^1^Department of Medical Science, Catholic University of the Sacred Heart, ‘A. Gemelli' University Hospital, Largo A. Gemelli, 8-00168 Rome, Italy; ^2^Endoscopy Unit, Catholic University of the Sacred Heart, ‘A. Gemelli' University Hospital, Largo A. Gemelli, 8-00168 Rome, Italy

## Abstract

*Background/Aim*. Uncomplicated diverticular disease (UDD) is a frequent condition in adults. The pathogenesis of symptoms remains unknown. Bacteria are able to interact with Toll-like receptors (TLRs) and to induce inflammation through both innate immunity and T-cell recruitment. We investigated the pattern of TLRs 2 and 4 and the intestinal homing in patients with UDD before and after a course of Rifaximin. *Methods*. Forty consecutive patients with UDD and 20 healthy asymptomatic subjects were enrolled. Among UDD patients, 20 were assigned to a 2-month course of treatment with Rifaximin 1.2 g/day for 15 days/month and 20 received placebo. Blood sample and colonic biopsies were obtained from patients and controls. The samples were collected and analyzed at baseline and at the end of treatment. Flow cytometry was performed using monoclonal antibodies (CD3, CD4, CD8, CD103, TCR-gamma/delta, CD14, TLR2, and TLR4). *Results*. In UDD, TLR2 and TLR4 expression on immune cell subpopulations from blood and mucosa of the affected colon are altered as compared with controls. Rifaximin treatment induced significant modifications of altered conditions. *Conclusions*. Our data show the role of TLRs in the development of inflammation in UDD. TLRs distribution is altered in UDD and these alterations are reversed after antibiotic treatment. This trial is registered with ClinicalTrials.gov: NCT02068482.

## 1. Introduction

Colon diverticulosis is a frequent condition in adults in Western countries and several patients experience clinical symptoms even when diverticulosis is not complicated by diverticulitis. This condition is being referred to as uncomplicated diverticular diseases (UDD) [1]. UDD symptoms pathogenesis remains unknown, but alterations in the diet and gut microbiota may be involved and could be responsible for “chronic low mucosal inflammation” [2]. Intestinal motility exerts a major control on gut microflora through the sweeping of luminal contents [3]. An alteration of the intestinal motility, especially decreasing anaerobic bacteria, can influence intestinal inflammation and be beneficial in both clinical and experimental models of colitis [4]. Rifaximin is beneficial to mice colitis [5]. Animal data indicate that a reduced load of bacteria may be useful in the prevention of colitis in susceptible individuals [5]. Although there is no supporting evidence from placebo-controlled trials, recommendations for management of acute episodes of UDD include medical treatment with broad-spectrum antibiotics [6, 7]. We have recently shown that both central and mucosal immunity are altered in UDD with increased recruitment of CD103 lymphocytes; treatment with Rifaximin ameliorates clinical symptoms (when present) and reduces CD103 levels, suggesting decreased mobilization of mucosal homing [8].

Rifaximin is an antibiotic that acts locally in the gastrointestinal tract with a broad spectrum of antibacterial activity. Fecal concentrations of Rifaximin are known to largely exceed the minimum inhibitory concentration values of pathogenic enteric bacteria. Rifaximin, instead of other systemic absorbed antibiotics, acts in the gastrointestinal tract modifying the gut microbiota (at the concentrations used, its action is mainly directed to pathobionts and much less to physiological gut flora). Furthermore, at the same time, Rifaximin, being a nonabsorbed antibiotic, has only little systemic effects [9].

To better understand the mechanisms involved, we reasoned that Rifaximin treatment may reverse immune system alteration by reducing bacteria related activation. Bacteria activate the immune system through specific receptors referred to as Toll-like receptors (TLRs) [10]. Bacterial lipopeptides (BLP) and lipopolysaccharides (LPS) are recognized by TLR2 and TLR4, respectively.

TLRs are involved in the generation of innate and adaptive immunity [11, 12]. Recent studies have shown that T cells also express certain types of TLRs [13, 14]. These TLRs can function as costimulatory receptors that complement T cell receptor- (TCR-) induced signals to enhance effector T cell activation [15]. Thus, TLRs also participate in adaptive immune response. No data have been reported on TLRs in humans affected by UDD. We therefore investigated TLR2, TLR4, and intestinal homing in UDD before and after a course of Rifaximin.

## 2. Material and Methods

The local Ethics Committee approved the study protocol and a written informed consent was obtained according to the principles of the Declaration of Helsinki (1983).

### 2.1. Patients' Recruitment

Over a period of 6 months, 40 consecutive patients with UDD (abdominal pain in the lower abdominal quadrant and change in bowel habit) and 20 healthy asymptomatic subjects undergoing screening colonoscopy for colorectal cancer were enrolled. The 40 patients with UDD were randomly assigned into two groups:

- 20 patients with UDD were assigned to a 2-month treatment with Rifaximin 1.2 g/day for 15 days/month.
- 20 patients with UDD on the same time received* placebo*.

Colonoscopy with multiple biopsies and blood samples were taken from patients and controls and repeated in patients at the end of the 2-month Rifaximin treatment or* placebo* course. Control healthy group was matched for gender, age, and body mass index (BMI) to the other two groups of patients.

Inclusion criteria in UDD patients group were as follows: endoscopic evidence of extensive diverticula limited to the sigmoid and descending colon; continuous gastrointestinal symptoms for at least 3 months; and age ≥18 years.

Exclusion criteria were as follows: inflammatory bowel diseases (IBD); mucosal inflammation at endoscopy and histology; present or past episodes of acute diverticulitis; colonic surgery; intestinal and extraintestinal cancer; use of antibiotics, anti-inflammatory drugs, probiotics, PPI, steroids, or fibers in the previous 12 weeks; hematological diseases; and pregnancy.

Acute diverticulitis was excluded on the basis of clinical signs and laboratory data.

Each patient underwent an abdominal ultrasonography to exclude abdominal masses or abscesses. Patients' identification was performed by the presence of left colonic diverticula, whilst controls had a normal colon.

### 2.2. Colonic Mucosa

Bioptic tissue samples were incubated with 100 UI/mL of human recombinant interleukin-2 (IL-2) to allow lymphocyte growth as previously described [8]. When tissue infiltrating lymphocytes (TILs) reached a sufficient number to perform the immunophenotype, surface markers were studied by immunofluorescence.

### 2.3. Peripheral Blood

Peripheral blood mononuclear cells (PBMC) were isolated by Ficoll-Hypaque according to standard procedures.

Whole blood cells (WBC), for granulocytes analysis, were isolated from EDTA-treated blood through red cell lysis. Briefly, 1 mL of venous blood was incubated with 1 mL of PBS and 10 mL of isotonic NH_4_Cl solution (155 mM NH_4_Cl, 10 mM KHCO_3_, 0.1 mM EDTA, and pH 7.4) for 10 min. Next, after two washes in PBS, the cells were stained as described below.

### 2.4. Flow Cytometry

PBMC, WBC, and TILs were stained for 30 min at 4°C with the following anti-human monoclonal antibodies (mAb): PE-Cy7 or PE conjugated-CD3 (clone SK7), PE-Cy7 or PE conjugated-CD4 (clone SK3), PE-Cy7 or PE conjugated-CD8 (clone SK1), FITC conjugated-CD103 (clone Ber-ACT8), PE conjugated-TCRgd (clone 11F2), PE-Cy7 conjugated-CD14 (clone M5E2), Alexa 482 conjugated-TLR2 (clone 11G7) (all from BD, San Diego, CA), and APC conjugated-TLR4 (clone HTA125) (eBiosciences, San Diego, CA).

### 2.5. Gating Strategy

Each analysis was performed using at least 100.000 cells that were gated in the region of the lymphocyte-monocyte population or in granulocytes population, as determined by light-scatter properties (forward scatter versus side-scatter).

Flow analysis was performed by a standardized 3- or 4-colour analysis protocol that was gated on cell stained with one fluorochrome, followed by 2-colour analysis of cells stained with the prescribed remaining fluorochromes.

Quadrants of dot plot were set using appropriate isotype controls for each antibody. Appropriate fluorochrome-conjugated isotype-matched mAbs (Beckman Coulter) were used as control for background staining in each flow acquisition. In these assays, careful colour compensation was performed before cell analysis. Expression of TLRs in lymphocytes was performed analysing at least 500 events. Mean fluorescence intensity (MFI) was calculated only for positive events after subtraction of specific isotype control MFI. All samples were analyzed with a FACS Calibur and CellQuest software (BD, Franklin Lakes, NJ).

### 2.6. Statistical Analysis

The statistical analysis was carried out using the GraphPad 6.0 statistical package. The Mann-Whitney* U* test was applied to the continuous variables, the changes observed in unpaired groups, and the Wilcoxon test in paired groups.

The Kruskal-Wallis test* H* was applied to evaluate whether significant differences exist between different samples and also for comparisons between independent samples. When appropriate, the correction for multiple comparisons was applied to the statistical significance.

## 3. Results

Demographic and clinical features of UDD patients and controls are summarized in Table 1. No statistical differences in smoking habits, alcohol daily intake, and body mass index (BMI) were observed between the two groups.

Good compliance to Rifaximin (>90% of the appropriate dose) was observed in all patients. A statistically significant decrease in symptoms was observed in patients two months after treatment. No adverse events to the drug or complications were observed.

### 3.1. Expression of TLRs

**Figure 1:**
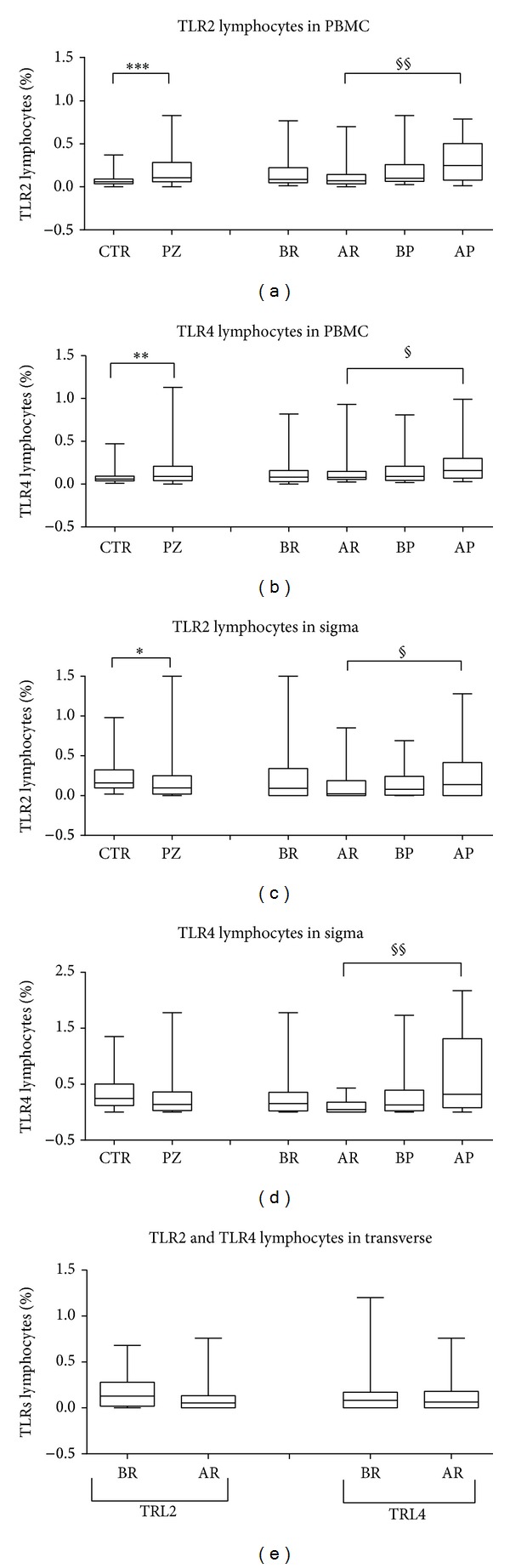
Expression of TLRs in lymphocytes. (a, b) TLR2 and TLR4 in peripheral blood lymphocytes, at baseline and after Rifaximin and placebo treatments. (c, d) TLR2 and TLR4 in sigma mucosa lymphocytes at baseline and after Rifaximin and placebo treatments. (e) TLR2 and TLR4 in transverse mucosa lymphocytes at baseline and after Rifaximin treatment. The values reported in box-and-whiskers plots show the minimum, the 25th percentile, the median, the 75th percentile, and the maximum. In particular, the whiskers go down to the smallest value (minimum) and up to the largest one (maximum), the top and bottom of the box are the 25th and 75th percentiles, and the line in the middle of the box is the median corresponding to 50th percentile (***P < 0.001, **P < 0.01, *P < 0.05 patients versus control (health or baseline)); ^§§^ *P* < 0.01, ^§^ *P* < 0.05 between treatments. Healthy control (CTR), UDD patients (PZ), before Rifaximin (BR), after Rifaximin (AR), before placebo (BP), and after placebo (AP).

In peripheral blood, at baseline, both the percentages of TRL2 and TRL4 lymphocytes were significantly higher (*P* = 0.0004 and *P* < 0.0096, resp.) in patients than in controls (Figures 1(a) and 1(b)).

After Rifaximin treatment (AR), the percentages of TRL2 and TRL4 lymphocytes in patients remained similar to basal values obtained before Rifaximin treatment (BR). However, TLR2 and TLR4 increased significantly (*P* = 0.0003 and *P* = 0.0104, resp.) in patients who received placebo treatment (after placebo or AP), as compared with patients who received Rifaximin treatment (Figures 1(a) and 1(b)). These data suggest that, in the lack of antimicrobial treatment, patient with UDD experiences an increase of TLRs expression.

In the sigmoid mucosa, at baseline, the percentages of TRL2 lymphocytes were lower (*P* = 0.0091) in patients than in controls, while TLR4 showed no significant differences (Figures 1(c) and 1(d)). In patients after Rifaximin, in a pattern similar to that observed in the peripheral blood, patients expressed significantly reduced percentages of both TLR2 and TLR4 (*P* = 0.0424 and *P* = 0.0022, resp.) in comparison to values observed in placebo treated patients (Figures 1(c) and 1(d)). Our data suggest that Rifaximin treatment contributes to maintain stable levels of TLRs and this is possibly related to the control of intestinal microflora.

Since the transverse mucosa of patients with UDD is supposed to be normal, lymphocytes in the transverse mucosa were studied as controls. In fact, no significant differences were observed before and after Rifaximin treatment (Figure 1(e)).

For a better understood role of TLR2 and TLR4 we also analyzed the subpopulations CD4 and CD8 lymphocytes as reported in Table 2.

**Figure 2:**
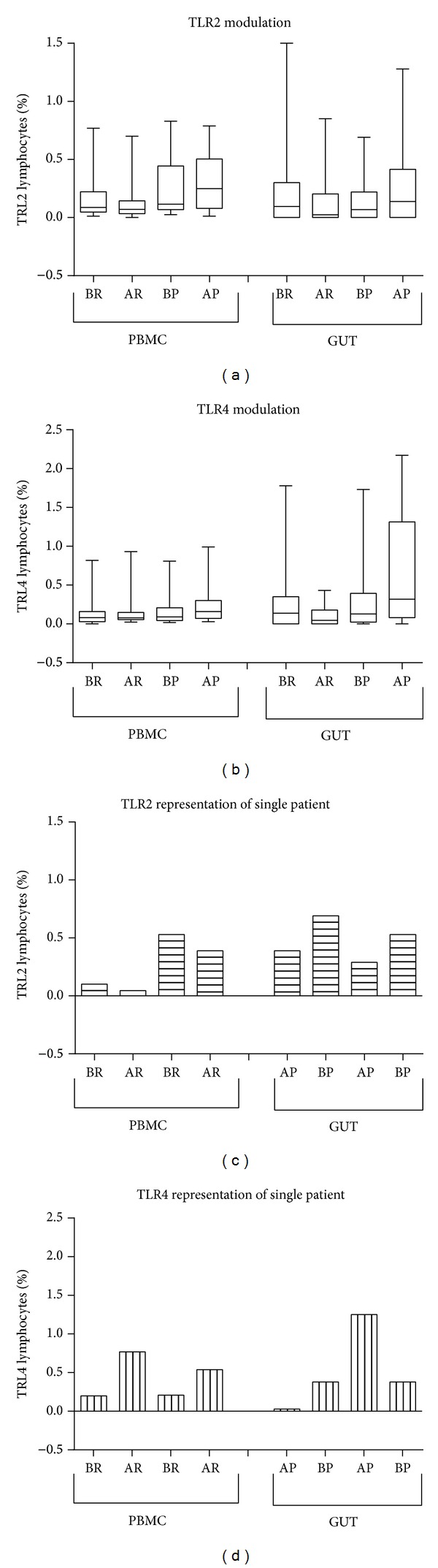
Modulation of TLR2 and TLR4 lymphocytes in PBMC and gut. (a, b) The comparison of modulation of TLR2 and TLR4 expression in the different patient groups between PBMC and gut (before and after placebo or Rifaximin). (c-d) Modulation of TLR2 and TLR4 expression observed in a single representative patient. The values reported in box-and-whiskers plots show the minimum, the 25th percentile, the median, the 75th percentile, and the maximum. In particular, the whiskers go down to the smallest value (minimum) and up to the largest one (maximum), the top and bottom of the box are the 25th and 75th percentiles, and the line in the middle of the box is the median, corresponding to 50th percentile, evaluated before Rifaximin (BR), after Rifaximin (AR), before placebo (BP), and after placebo (AP).

TLR2 and TLR4 were analyzed in PBMC and sigma mucosa of same patient. To clarify the way in which the modulation of TLR2 and TLR4 in PBMC and sigma mucosa occurred, we compared the modulation of TLR2 and TLR4 expression between PBMC and gut in the different patient groups (BR and AR and BP and AP) (Figure 2). We observed the same trend of TLRs modulation in both the mucosa and the PBMC.

We observed coexpression of TRL2 and TLR4 on lymphocyte in both PBMC and sigma mucosa of the same patient. Moreover, we reported that the modulation of TLR2 and TLR4 correlated in PBMC (95% confidence interval 0.21 to 0.447; *r* = 0.2366; *P* < 0.05) and in transverse mucosa (95% confidence interval 0.052 to 0.687; *r* = 0.4195; *P* < 0.05), but not in sigma mucosa (95% confidence interval −0.334 to 0.256; *r* = −0.043; *P* < 0.05).

The phenotype of TLR lymphocytes was also evaluated for the CD4 and CD8 lymphocytes subpopulations to better understand the effect of Rifaximin in UDD patients; the results are summarized in Table 2. We show that both TLR2-CD8 and TLR2-CD4 cells were increased in patients PBMC as compared to controls. In the sigma mucosa, after Rifaximin, TLR4-CD4 cells were significantly reduced.

**Figure 3:**
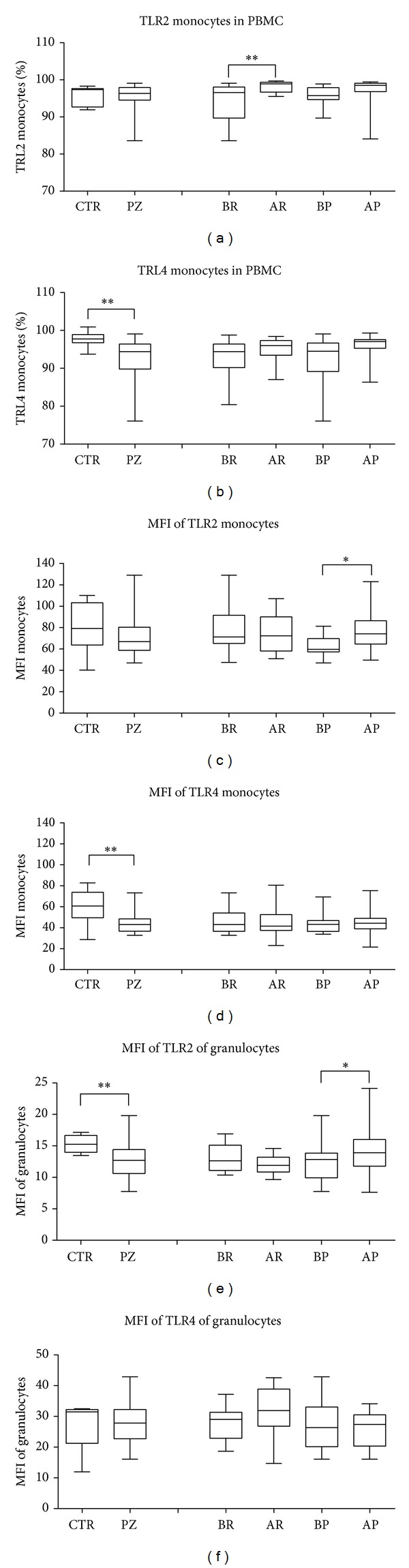
Expression of TLRs in monocytes and granulocytes. (a, b) Percentage of TLR2 and TLR4 monocytes in peripheral blood, at baseline and after Rifaximin and placebo treatments. (c, d) Median of fluorescence (MFI) of TLR2 and TLR4 monocytes in peripheral blood at baseline and after Rifaximin and placebo treatments. (e, f) MFI of TLR2 and TLR4 granulocytes in peripheral blood at baseline and after Rifaximin and placebo treatments. The values reported in box-and-whiskers plots show the minimum, the 25th percentile, the median, the 75th percentile, and the maximum. In particular, the whiskers go down to the smallest value (minimum) and up to the largest one (maximum), the top and bottom of the box are the 25th and 75th percentiles, and the line in the middle of the box is the median corresponding to 50th percentile (***P < 0.001, **P < 0.01, and *P < 0.05 patients versus controls (health or baseline)). Healthy control (CTR), UDD patients (PZ), before Rifaximin (BR), after Rifaximin (AR), before placebo (BP), and after placebo (AP).

In peripheral blood, at baseline, no changes were found in TRL2 monocytes (CD14+) percentages. The percentages of TRL4 monocytes were lower (*P* = 0.0019) in patients with respect to controls (Figures 3(a) and 3(b)). After Rifaximin treatment the percentages of TRL2/CD14+ increased significantly (*P* = 0.0039) with respect to before Rifaximin (Figure 3(a)); no changes were found in TRL4 monocytes percentages (Figure 3(b)).

Analysis of MFI showed that, in monocytes, the TLR4 MFI baseline was significantly lower than control (*P* = 0.0014), while after Rifaximin treatment no change was found (Figure 3(d)). On the other hand, TLR2 MFI was increased after placebo (*P* = 0.0238), while remained unchanged in the after Rifaximin patients. In granulocyte populations, at baseline TRL2 MFI decreased (*P* = 0.0029), while TLR2 MFI increased after placebo (*P* = 0.0398) (Figure 3(e)). All these findings suggest that Rifaximin treatment contributes to maintain stable levels of TLRs and possibly related to the control of intestinal microflora.

We did not find any correlation between TLRs expression in lymphocytes and monocytes; instead, we found correlations for both TLR2 and TLR4 in monocytes and granulocytes; in fact, the direct associations were found between MFI monocytes and MFI granulocytes in UDD patients at T0 (TLR2 correlation coefficient: 0.530, *P* = 0.013; TLR4 correlation coefficient: 0.478, *P* = 0.033).

### 3.2. CD103 Lymphocytes

CD103 is a gut homing receptor present on the surface of lymphocytes and marks gut-homing recruitment of lymphocytes. We analyzed CD103 expression in peripheral blood and in sigmoid and transverse mucosa TILs.

**Figure 4:**
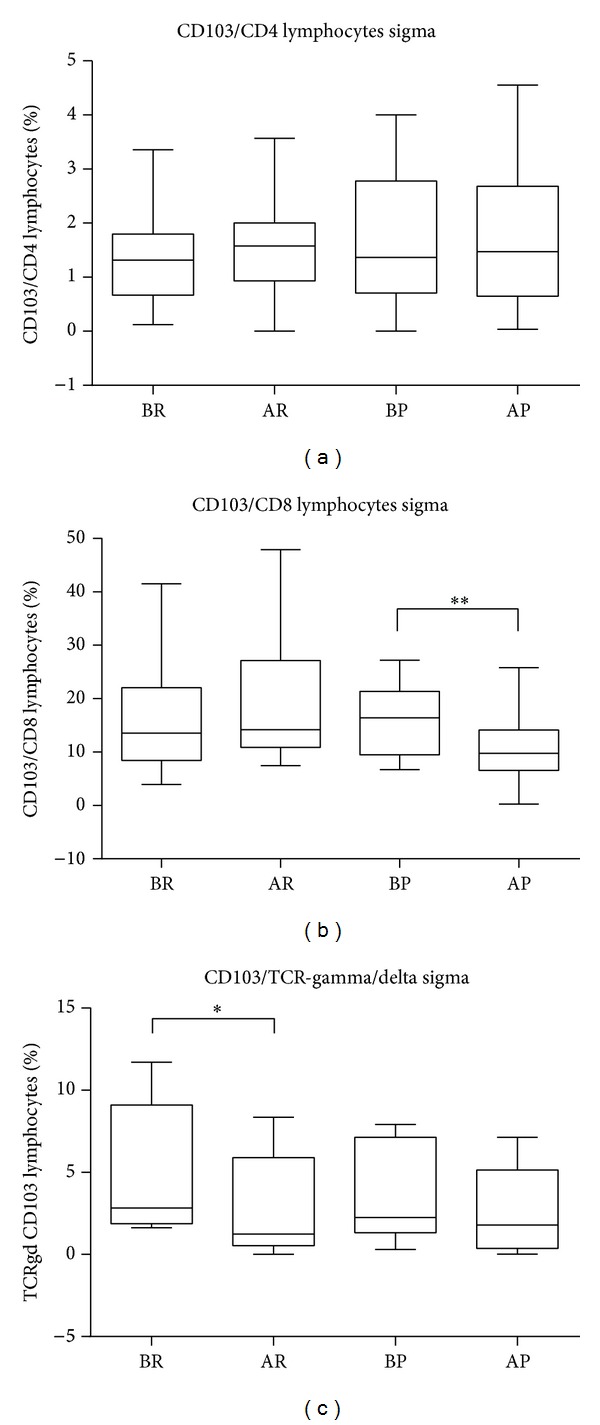
Expression of CD103 in lymphocytes of sigma mucosa. (a) Percentage of CD103 in CD4 lymphocytes subpopulation after Rifaximin and placebo treatments. (b) Percentage of CD103 in CD8 lymphocytes subpopulation after Rifaximin and placebo treatments. (c) Percentage of TCR-gamma/delta CD103 lymphocytes subpopulation after Rifaximin and placebo treatments. The values reported in box-and-whiskers plots show the minimum, the 25th percentile, the median, the 75th percentile, and the maximum. In particular, the whiskers go down to the smallest value (minimum) and up to the largest one (maximum), the top and bottom of the box are the 25th and 75th percentiles, and the line in the middle of the box is the median corresponding to 50th percentile (*P < 0.05 before versus after treatments in patients); before Rifaximin (BR), after Rifaximin (AR), before placebo (BP), and after placebo (AP).

Considering both mucosal CD4 and CD8/CD103 cells, no significant variations were observed between patients and controls in colonic tissue (data not shown). In peripheral blood, in UDD patients, we found an increased recruitment of CD103 lymphocytes (as reported in our previous work (8)). When we evaluated CD4/CD103 and CD8/CD103 in sigma mucosa before and after Rifaximin treatment, we did not find any change. However, in patients treated with placebo the percentage of CD8/CD103 lymphocytes was lower after placebo (*P* = 0.0072) than before placebo (Figure 4(b)). This suggests that Rifaximin may keep a constant homing flux to the sigma of the CD8 cells.

The gamma/delta T cells are abundant in the gut mucosa. We therefore evaluated these cells in combination with the homing marker CD103 in TILs derived from sigma of UDD patients.

After treatment with Rifaximin the percentages of the sigma CD103 TCR-gamma/delta lymphocytes decreased significantly (*P* = 0.0182) with respect to the before Rifaximin percentages, while no differences were found in patients treated with placebo (Figure 4(c)).

No correlations were found between TLRs and CD103 expression in TCR-gamma/delta lymphocytes.

## 4. Discussion

We have investigated bacterial ligands TLR2 and TLR4 and other markers in peripheral blood and mucosa and on lymphocytes, monocytes, and granulocytes of patients with UDD before and after antibacterial treatment. Several abnormalities of TLRs can be reversed by Rifaximin treatment.

We showed that UDD induces significant modifications of TLR2 and TLR4 expression on several immune system cell subpopulations isolated from both peripheral blood and affected mucosa as compared with controls. Since TLR2 and TLR4 are receptors for ligands present on bacterial walls, we reasoned that antibiotic treatment could reverse colonic inflammation associated with UDD. Rifaximin treatment induced significant modifications of several altered conditions: either restoring the values observed in controls, or limiting the deviations from normal range observed after 2-month placebo treatment. Diverticula, even when not acutely inflamed, represent an anatomical abnormality where excessive growth of bacteria occurs. This may lead to clinical symptoms. We have previously shown that both clinical symptoms and immunological abnormalities can be ameliorated by a short course of Rifaximin [8].

TLR2 and TLR4 are not constitutively expressed on human T cell surface, but their expression requires activation of T cells by TCR complex [16]. We have previously reported increased CD25+ cells in UDD peripheral blood [8]. Accordingly, we showed that TLR2 and TLR4 lymphocytes in the peripheral blood are increased in patients versus controls suggesting more activated circulating T cells in peripheral blood.

TLRs are involved in the activation of NF-kB [17]. Moreover, NF-*κ*B is a critical factor in antibacterial defense [18] and activates the secretion of proinflammatory cytokines resulting in a Th1 response [19]. Thus, Rifaximin keeping under control the activation of TLRs may also limit the triggering of Th1 adaptive immune response. These findings suggest that Rifaximin also has a systemic action on immune system.

In the sigmoid mucosa, percentages of TLR2 and TLR4 lymphocytes are decreased in patients and remain stable after Rifaximin, while we observed an increase of these populations after placebo. It may be suggested that in UDD immunotolerance to commensal bacteria and short-term TLRs activation impairment might be related to increased inflammation that leads to the development of symptoms in UDD. No differences were observed in modulation of TLR2 and TLR4 lymphocytes between PBMC and sigma mucosa. However, data showing that TLR2 and TLR4 increase in time in after placebo patients suggest that the TLRs defect is not constitutive, but it is limited to the stage of early activation. This mechanism can be the result of a lack of lymphocytes activation since TLR2 is expressed in activated cells [16]. Alternatively, lymphocytes in UDD patients recognize the antigens through adaptive immune response, which requires time to be activated, rather than through innate immunity. Thus, our results of reduced TLR2 expression in sigmoid mucosa could be explained by the delayed activation. In fact, in after placebo patients TLR2 expression is increased. It is interesting to note that we observed a reduced TLR2 expression only in TILs and not in peripheral blood. Our data are similar to evidences in the pleural fluid of patients with tuberculosis infection [19] and filariasis [20], suggesting that downregulation of TLRs at the site of infection and not in the periphery may be connected with a reduction in the secretion of proinflammatory cytokines [19]. Alternatively, an involvement of the T regulatory cells (Tregs) could be proposed [21]. The agonists of TLRs may enhance Tregs proliferation, rendering Tregs transiently nonsuppressive [22].

Finally, we have shown as Rifaximin controls the activation of TLRs in TILs. This may limit the triggering of Th1 adaptive immune response. Limiting the activation of Th1 immune response, involved in the pathogenesis of several inflammatory diseases [23, 24], may be a mechanism by which Rifaximin acts as an anti-inflammatory agent in addition to its antibiotic effect [25].

We have also shown that both TLR2-CD8 and TLR2-CD4 cells were increased in patients PBMC as compared to controls. We hypothesize that, as in UDD bacterial infections are more frequent, lymphocytes expressing TLR2 and TLR4 are already mobilized and ready to mount the immune-response. After Rifaximin, TLR-CD4 cells are significantly reduced in sigmoid mucosa, thus confirming the indirect role of the antibiotic on mucosal immunity. In fact, the reduced number of TLR4-CD4 lymphocytes may be related to the fact that, after antibiotic treatment, there is a reduced need of TLR4-CD4 cells which are important in immune responses against bacteria. CD4 cells are instrumental in starting immune response to produce specific antibodies, cell to cell cross talking through production of cytokines, delivering activation signals and induction of activation markers. The TLR2-CD4 cells are also reduced, but this reduction is not significant. This suggests a similar trend for both TRL2-CD4 and TLR4-CD4 cells. However our data show that Rifaximin prevalently acts on TLR4-CD4 cells. Only further studies may clarify the different responses of CD4 cells to the antibiotic.

Monocytes and granulocytes are essential cells in the innate immune response and reflect the level of inflammation [26]; we therefore analyzed TLRs expression in peripheral blood monocytes and granulocytes.

In monocytes, TLR4 was decreased in peripheral blood both as percentages of cells and MFI, in patients versus controls. These conditions were not modified by Rifaximin. Then, we hypothesized that a defect in TLR4 expression predisposes to diverticular disease by impairing antibacterial defense. This could be related to genetic polymorphism of TLR4 as described by other authors [27, 28]. This reinforces the possibility that, in UDD, the increased bacterial load may contribute to the development of symptoms related to UDD.

Instead, both percentages and MFI of TLR2 monocytes in patients were similar to controls. After Rifaximin TLR2 monocytes were increased in percentage, while TLR2 expression was stable. These results suggest that Rifaximin limits the increase of TLR2 expression, probably in relation to its bactericide action on pathogenic flora. This was reinforced by the increase of MFI expression after placebo, both in monocytes and in granulocytes, which could be related to a stimulation of proinflammatory cytokine and TLR-ligands due to increased bacterial load. Our finding may reflect increased activation of monocytes, as reported [26].

Finally, CD103 cells are lymphocytes homing to the gut tissue [23]. Sigma's CD103 cells are normal in UDD (data not shown). After placebo, we observed a significant decrease of these cells, while after Rifaximin their percentage remained unchanged, suggesting that Rifaximin can maintain intestinal homing within the normal range. Moreover, the decrease of CD103 cells can be due to cell death, which may be related to insufficient recruitment.

Furthermore, we analyzed the involvement of gamma-delta T cells both in peripheral blood and in tissue. Gamma-delta T cells exert a regulatory function. These cells are a minor population in the peripheral blood but constitute a major population among intestinal intraepithelial lymphocytes [29]. Thus, we focused on gamma-delta T cells expressing the intestinal homing receptor CD103 [23].

After Rifaximin, mucosal CD103 positive gamma-delta T-cells were reduced and our data suggest that homing gamma-delta cells directly correlate with gut inflammation in UDD and their reduction supports the anti-inflammatory activity of Rifaximin either through the bacterial load reduction or because Rifaximin has been showed to have a direct anti-inflammatory activity, as already reported above. The gamma-delta T cells may be considered as a marker of inflammation, similar to Tregs, as reported in other papers of our group [21, 23, 30]. While our data clearly show the reduction of inflammatory-related features after Rifaximin, only further studies will demonstrate if these effects are due to reduced bacterial growth or due to intrinsic anti-inflammatory activity recently observed with Rifaximin [25]. It is interesting to note that, in the mice, gamma-delta activation is associated with Th17 cells acting through ligation of TLR2 and TLR4 [31].

In summary, we have described a vast immunological pattern of several innate and adaptive cells markers including TLRs, which may be involved in the pathogenesis and clinical course of the diverticular disease, and we have showed its variations before and after the antibiotic therapy with Rifaximin. We suggest that TLRs are modified by the presence of pathogenic flora in UDD. The role of pathogenic flora is supported by the finding that Rifaximin acts in the gut mucosa homeostasis by limiting the activation of TLRs. Furthermore, Rifaximin may also have a luminal anti-inflammatory function modulating the adaptive immune response in an inhibitory sense. Rifaximin keeps under control TLRs expression in peripheral blood suggesting that, in addition to its activity in gut mucosa, it may also have a systemic action on immune system.

We have shown that, in UDD, TLRs and several immune cell populations are altered in patients with respect to controls, suggesting that in UDD inflammation is present even in the absence of severe clinical symptoms.

## Figures and Tables

**Table 1:** Demographic and clinical characteristics of UDD patients and controls.

	UDD patients (*n* = 40)	Controls (*n* = 21)
Gender (male/female)	21/19	5/16
Age, median (range), years	67.5 (39–84)	53 (20–78)
Smoking habit (yes)	8	7
Alcohol intake (>60 g/day)	18	7
Weight, mean (range) (Kg)	70.38 (52–100)	66.14 (52–84)
Height, mean (range) (m)	1.66 (1.41–1.80)	1.67 (1.50–1.80)
Body mass index (range)	22.6 (17–24)	22 (17–22)

**Table 2:** TLRs phenotype in CD4 and CD8 lymphocytes subpopulation.

	PBMC				Sigma				Transverse			
	CD4 TLR2	CD8 TLR2	CD4 TLR4	CD8 TLR4	CD4 TLR2	CD8 TLR2	CD4 TLR4	CD8 TLR4	CD4 TLR2	CD8 TLR2	CD4 TLR4	CD8 TLR4
CTR	0.069 ± 0.016	0.094 ± 0.018	0.354 ± 0.206	0.090 ± 0.020	0.222 ± 0.048	0.250 ± 0.078	0.400 ± 0.11	0.356 ± 0.096	0.224 ± 0.03	0.255 ± 0.06	0.421 ± 0.10	0.45 ± 0.11
PZ	0.193 ± 0.041**	0.244 ± 0.037*	0.169 ± 0.035*	0.26 ± 0.059	0.191 ± 0.038	0.239 ± 0.069	0.371 ± 0.104	0.284 ± 0.078	0.171 ± 0.046	0.246 ± 0.10	0.308 ± 0.19	0.489 ± 0.28
BR	0.082 ± 0.015	0.276 ± 0.070	0.133 ± 0.042	0.233 ± 0.086	0.182 ± 0.055	0.258 ± 0.096	0.392 ± 0.17	0.285 ± 0.11	0.171 ± 0.05	0.246 ± 0.10	0.308 ± 0.19	0.489 ± 0.28
AR	0.115 ± 0.044	0.161 ± 0.045	0.197 ± 0.084	0.221 ± 0.074	0.092 ± 0.029	0.143 ± 0.057	0.076 ± 0.037*	0.194 ± 0.07	0.13 ± 0.06	0.232 ± 0.16	0.354 ± 0.21	0.488 ± 0.29
BP	0.251 ± 0.060	0.264 ± 0.058	0.205 ± 0.057	0.293 ± 0.082	0.371 ± 0.18	0.219 ± 0.101	0.592 ± 0.27	0.284 ± 0.12	ND	ND	ND	ND
AP	0.352 ± 0.084**	0.416 ± 0.081**	0.269 ± 0.066	0.293 ± 0.086	0.536 ± 0.21	0.419 ± 0.133*	1.482 ± 0.70*	1.442 ± 0.60	ND	ND	ND	ND

The values are reported as Mean ± SEM *P* < 0.001, **P < 0.01, *P < 0.05 patients versus control (health or baseline); *P* < 0.01, *P* < 0.05 between treatments. Healthy control (CTR), UDD patients (PZ), before Rifaximin (BF), after Rifaximin (AR), before placebo (BP), and after placebo (AP).

## Abbreviations

AP: After placebo
AR: After Rifaximin
BP: Before placebo
BR: Before Rifaximin
MFI: Mean fluorescence intensity
TILs: Tissue infiltrating lymphocytes
TLRs: Toll-like receptors
UDD: Uncomplicated diverticular diseases
WBC: Whole blood cells.
